# Effectively identifying regulatory hotspots while capturing expression heterogeneity in gene expression studies

**DOI:** 10.1186/gb-2014-15-4-r61

**Published:** 2014-04-07

**Authors:** Jong Wha J Joo, Jae Hoon Sul, Buhm Han, Chun Ye, Eleazar Eskin

**Affiliations:** 1 Bioinformatics IDP, University of California, Los Angeles, CA, USA; 2 Computer Science Department, University of California, Los Angeles, CA, USA; 3 Division of Genetics, Brigham & Women’s Hospital, Harvard Medical School, Boston, MA, USA; 4 Program in Medical and Population Genetics, Broad Institute of Harvard and MIT, Cambridge, MA, USA; 5 Broad Institute, 7 Cambridge Center, Cambridge, MA, USA; 6 Department of Human Genetics, University of California, Los Angeles, CA, USA

## Abstract

Expression quantitative trait loci (eQTL) mapping is a tool that can systematically identify genetic variation affecting gene expression. eQTL mapping studies have shown that certain genomic locations, referred to as regulatory hotspots, may affect the expression levels of many genes. Recently, studies have shown that various confounding factors may induce spurious regulatory hotspots. Here, we introduce a novel statistical method that effectively eliminates spurious hotspots while retaining genuine hotspots. Applied to simulated and real datasets, we validate that our method achieves greater sensitivity while retaining low false discovery rates compared to previous methods.

## Background

Expression quantitative trait loci (eQTL) mapping is an approach linking genetic variation to gene expression to identify genomic loci containing gene expression modulators. An interesting observation in previous eQTL studies is that expression levels of thousands of genes may be affected by genetic variation at a single location called a regulatory hotspot. Although some of the identified regulatory hotspots correspond to genetic variants that truly govern expression of many genes, it has been recently reported that spurious hotspots that do not have genetic effects on genes may appear due to confounding factors such as batch effects. In this paper, we introduce a novel statistical method that effectively eliminates spurious regulatory hotspots while retaining genuine hotspots resulting from true genetic effects.

Understanding the relationship between genetic variation and gene regulation has recently received significant interest. The most common approach for studying this relationship is through eQTL, where both genetic variation and expression levels are collected from a set of individuals and associations between genetic variation and expression are estimated [1-9]. Any identified association, or eQTL, suggests the presence of a region harboring genetic variation that affects expression levels.

In eQTL mapping, two types of eQTLs are analyzed: *cis*-eQTLs that are in close proximity to the gene locus and *trans*-eQTLs that occur at greater distances from the gene locus [10]. Previous eQTL studies for multiple organisms [2-4,6] have shown that many genes are *trans*-regulated by a small number of genomic regions, known as ‘regulatory hotspots’. Although several eQTL studies have successfully identified regulatory hotspots [11-13], it has been reported in studies of recombinant inbred mice that regulatory hotspots replicate poorly [14]. Previous studies have discovered that these regulatory hotspots are spurious associations caused by various confounding factors, such as batch effects or other technical artifacts, which induce noise during sample preparation or expression measurements [15-17]. Confounding factors create heterogeneity in expression data and may induce spurious associations between SNPs and gene expressions, leading to the identification of ‘spurious regulatory hotspots’ [18]. In these spurious hotspots, SNPs appear to be associated with gene expression levels, although they do not have genetic effects on the genes.

Several computational methods have been developed to correct for confounding effects using various statistical methods such as singular value decomposition or linear mixed models [18-21]. The main assumption behind most of these methods is that the confounding factors influence the global correlation structure between the gene expression values. Hence, the methods, such as Intersample Correlation Emended (ICE) [18] and SVA [19], attempt to estimate the global correlation structure and use it as a covariate in the association to remove confounding effects from the association statistic. Although these methods effectively remove spurious regulatory hotspots, they may also remove true hotspots caused by genetic factors. This is because the global correlation structure contains genetic effects, so by correcting for the global structure, genetic effects are removed as well. For example, in a well-studied yeast dataset, several hotspots are known to be true genetic effects since they have been validated by additional data such as protein measurements [22,23]. Unfortunately, these hotspots are removed in addition to the spurious ones. Other methods [20,21] also do not explicitly remove true genetic signals and either eliminated true hotspots or failed to remove spurious hotspots in our experiments.

In this paper, we introduce a new method called Next-generation Intersample Correlation Emended (NICE) eQTL mapping, which attempts to eliminate spurious regulatory hotspots while retaining hotspots caused by genetic effects by utilizing a novel statistical framework. Our method leverages an insight that confounding factors affect the majority of genes, while genetic effects only affect a subset. This insight allows us to distinguish between confounding and genetic effects. We used a recently developed statistic [24] to differentiate between genes that are affected by both genetic effects and confounding effects versus genes that are affected by only confounding effects. Using genes only affected by confounding, we are able to correct for the confounding effects but preserve the genetic effects. We first show by simulations that NICE successfully eliminates spurious regulatory hotspots while preserving regulatory hotspots corresponding to real genetic effects. On the other hand, previous methods either fail to eliminate confounding effects or fail to retain the genetic effects.

We demonstrated the utility of NICE with a yeast dataset. Versions of a yeast dataset were generated in 2005 [2] and 2008 [25]. Since they were generated 3 years apart in different locations, the hotspots that are shared between the datasets are likely to be the real genetic effects, while hotspots that are different between the datasets are likely to be spurious hotspots. We used our method on only the first dataset to see if we could discriminate between which hotspots are real and spurious as determined by the second dataset. Applied to the yeast dataset, NICE identified 83% (sensitivity) of the putative regulatory hotspots that are consistent between the two versions of the yeast dataset. Previous methods applied to this dataset either eliminated many of the putative hotspots or predicted many spurious hotspots. In addition, NICE eQTL mapping identified either more or a comparable number of *cis* associations relative to previous methods. Furthermore, applied to a yeast dataset grown in different conditions, NICE identified genes that are related to gene–environment interactions and discovered novel yeast regulatory hotspots that are likely to have a true biological mechanism.

## Results

### NICE eQTL mapping

**Figure 1:**
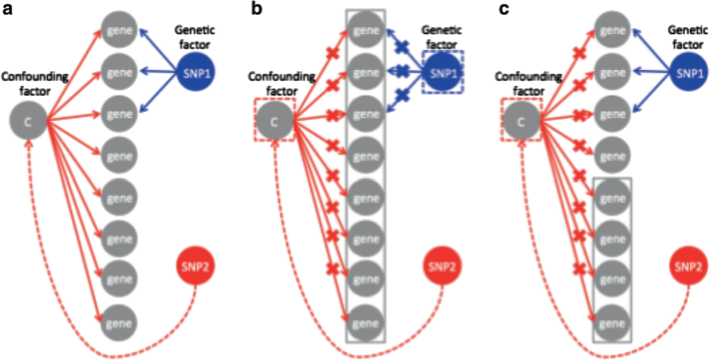
**GraphicalFigures: format model of ICE and NICE with correction for confounding effects. (a)** Genetic and spurious associations. SNP 1 has a genetic effect on the first three genes (blue arrows). SNP 2 has no genetic effect on any of the genes; however, it has spurious associations with many of the genes, because, by chance, it happens to be correlated with a confounding factor (red arrows). **(b)** ICE [18] models the confounding effects by estimating the global correlation structure of the expression levels of all genes (gray block). However, this eliminates genetic associations in addition to confounding effects since any regulatory hotspots, such as the first three genes, will also be captured in the global correlation structure and be eliminated. **(c)** NICE uses only a subset of genes to model the global correlation structure between expression levels used to correct for confounding factors. Since any subset of genes will capture the confounding effects, by using the bottom four genes (gray block) we can eliminate the confounding effects but preserve the genetic effects. SNP, single nucleotide polymorphism.

Our goal was to identify true genetic associations in an eQTL mapping study without predicting spurious associations due to confounding factors. Consistent with previous approaches that correct for the confounding factors based on singular value decomposition or linear mixed models, we assumed that the confounding factors affect the global correlation structure of expressions. That is, we assumed that confounding factors affect the expression levels of most of the genes. On the other hand, we assumed that genetic factors only affect the expression levels of a subset of the genes related to the regulatory pathways. Figure 1(a) shows a graphical model that contains both genetic and spurious associations due to a confounding factor. SNP 1 has a genetic effect on multiple genes and thus, is a regulatory hotspot. Unlike SNP 1, SNP 2 has no direct genetic effects on any of the genes. However, SNP 2 has spurious associations with many of the genes because, by chance, it happens to be correlated with the confounder and this results in a spurious regulatory hotspot. 

To eliminate spurious associations, ICE [18] models the confounding effects by estimating the global correlation structure of the expression levels of all genes and uses this structure as a covariate in the association statistic. This has the same effect as if the confounding factor itself is included as a covariate in the association statistic, removing its effect. Unfortunately, any regulatory hotspots, as in the case of the first three genes of Figure 1(b), will also be captured in the global correlation structure and be eliminated. For this reason, ICE [18] tends to eliminate true regulatory hotspots in addition to confounding effects.

In contract to ICE [18], NICE uses only a subset of genes to model the global correlation structure between expression levels used to correct for confounding factors. Since most genes are affected by confounding effects, any subset of genes will likely capture the confounding effects and utilizing only those genes to estimate the global correlation structure is enough to correct for the confounding factor. Theoretically, if we can correct for confounding effects using the genes that are not involved in true regulatory hotspots, we would eliminate spurious associations while preserving true genetic associations. Unfortunately, we do not know in advance which genes are involved in the regulatory hotspots, which complicates the choice of which genes to use to correct for the confounding. Practically, almost all genes are affected by confounding effects while only some genes have both confounding and genetic effects. We expect this second group of genes to show stronger associations than the others. Thus we use the weakly associated genes to model the confounding factors. For example, in Figure 1(c), by using the four most weakly associated genes, we can correct for the confounding effects but preserve the genetic effects.

### NICE eliminates spurious regulatory hotspots while preserving true genetic effects

To validate that our method eliminates spurious regulatory hotspots while preserving regulatory hotspots corresponding to real genetic effects, we generated a simulated dataset with both true regulatory hotspots and a batch effect that creates spurious hotspots. We created a dataset that has 100 samples with 1,000 SNPs and 1,000 gene expression levels. We added five *trans*-regulatory hotspots and *cis* effects. For each of the *trans*-regulatory hotspots, 20% of the genes have *trans* effects. SNPs were randomly generated with minor allele frequencies of 30%. A batch effect was simulated where expression levels in the first half of the samples were correlated with each other, but not correlated with the second half of the samples, and vice versa.

**Figure 2:**
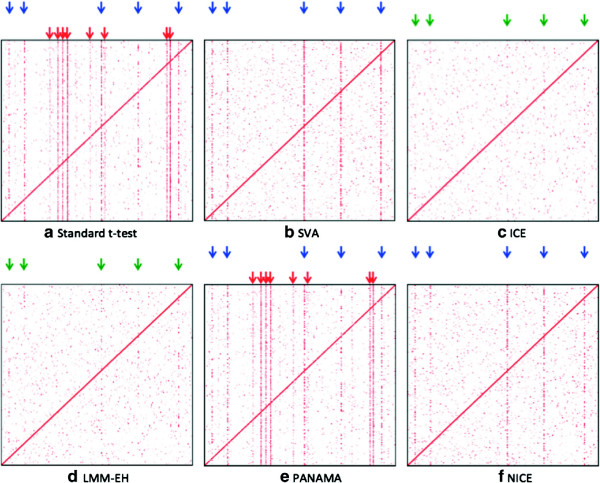
**eQTL maps for different methods applied to the simulated data.** The *x*-axis corresponds to SNP positions and the *y*-axis corresponds to the gene positions. The intensity of a point on the plot represents the significance of the association. The diagonal band represents the *cis* effects and the vertical bands represent hotspots. Blue arrows show the locations of real genetic regulatory hotspots, green arrows indicate missing hotspots and red arrows show spurious hotspots. The eQTL map for the standard *t*-test **(a)**, SVA **(b)**, ICE **(c)**, LMM-EH **(d)**, PANAMA **(e)** and NICE **(f)** are shown.

We visualized the results of the eQTL study with an eQTL plot such as those shown in Figure 2. The *x*-axis corresponds to SNP positions and the *y*-axis corresponds to the gene positions. The intensity of a point on the plot represents the significance of the association. The diagonal band represents the *cis* effects and the vertical bands represent hotspots. On the eQTL plot, we mark successfully identified regulatory hotspots with blue arrows, missed regulatory hotspot with green arrows and spurious hotspots with red arrows. In the simulated data, the eQTL plot has five regulatory hotspots (Figure 2(a), blue arrows) and eight spurious hotspots (Figure 2(a), red arrows) induced by the batch effect we simulated.

**Figure 3:**
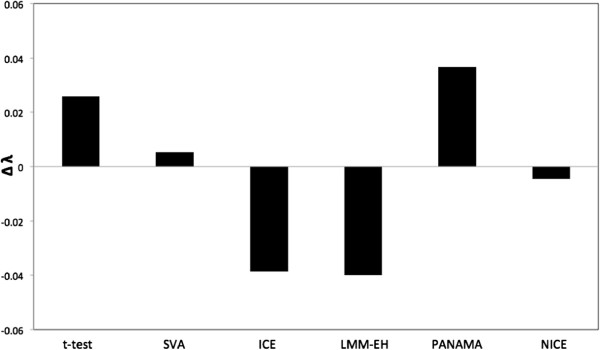
**Inflation factors for different methods for simulated data.** Δλ is defined as 1−λ.

We compared our method with several methods including SVA [19], ICE [18], LMM-EH [20] and PANAMA [21]. They were all used to correct expression heterogeneity on the simulated data and the results are shown on eQTL plots (Figure 2). NICE successfully identified five real regulatory hotspots and eliminated spurious ones (Figure 2(f)). SVA also identified five real regulatory hotspots and eliminated spurious ones (Figure 2(b)), which was expected as SVA is designed to capture only the broad signal and our simulated data contains only one large batch effect. In the next section, we show that SVA does not perform as well on a real dataset that contains more realistic confounding effects. On the other hand, ICE and LMM-EH removed not only spurious hotspots but also real hotspots (Figure 2(c),(d)). PANAMA failed to remove the spurious hotspots and did not show a big difference with the standard *t*-test (Figure 2(a),(e)). The results are summarized in Table 1. Given the number of real (*R*), missing (*M*) and spurious (*S*) hotspots identified, we calculated the sensitivity and false discovery rate (FDR) as *R*/(*R*+*M*) and *S*/(*R*+*S*), respectively. All methods successfully identified *cis* effects. We further studied the test statistics of *P* values by estimating the genomic control inflation factor λ[26] to check if the *P* values are either inflated (λ>1) or deflated (λ<1). Figure 3 shows Δ λ, which is defined as 1−λ. The Δ λ values of SVA and NICE are close to zero. On the other hand, the standard *t*-test and PANAMA show inflation (Δ λ>0) and ICE and LMM-EH show deflation (Δ λ<0).

**Table 1:** Number of real, missing and spurious hotspots identified by different methods applied to simulated data

**Method**	**Real hotspots**	**Missing hotspots**	**Spurious hotspots**	**Sensitivity**	**False discovery rate**
*t*-test	5	0	8	1.0	0.62
SVA	5	0	0	1.0	0
ICE	0	5	0	0	N/A
LMM-EH	0	5	0	0	N/A
PANAMA	5	0	8	1.0	0.62
NICE	5	0	0	1.0	0

N/A, not applicable.

### NICE eliminates spurious hotspots while preserving genetic hotspots for a yeast dataset

We took advantage of a unique dataset consisting of two versions of a yeast dataset generated in 2005 [2] and 2008 [25] to validate our method. The two datasets contain similar strains, but were generated 3 years apart in different locations. For this reason, the hotspots that are shared between the datasets are likely real genetic effects, while hotspots that are different between the datasets may be spurious hotspots caused by technical confounding factors present at the time of generation of the datasets. In addition, some of these hotspots were further validated by other experimental data such as protein levels [22,23].

**Figure 4:**
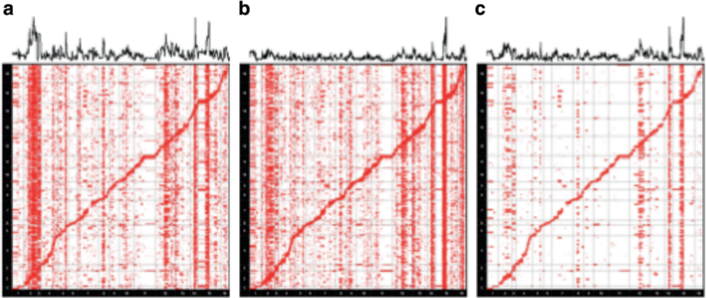
**eQTL maps of two versions of a yeast dataset that were generated 3 years apart in different locations.** The standard *t*-test was used to generate the *P* values. **(a)** eQTL map of the yeast dataset generated in 2005 [2]. **(b)** eQTL map of the yeast dataset generated in 2008 [25]. **(c)** eQTL map using the maximum *P* value of the two datasets to show the putative genetic associations in the yeast dataset. The graph on the top of each eQTL map shows the strength of regulatory hotspots as the average over all genes of the − log*P* values for a given SNP.

To determine which hotspots in the two datasets are regulatory hotspots due to genetic effects, we used the following approach. We first computed a *P* value for each gene-SNP pair in both datasets using the standard *t*-test (Figure 4(a),(b)). We then merged the *P* values of the two datasets by taking a maximum *P* value between the two (Figure 4(c)). The idea is that associations due to true genetic effects are likely to have significant *P* values in both datasets while associations due to the confounding effects tend to have a significant *P* value in only one of the datasets. Thus, by taking the maximum *P* value, we can identify the associations that are significant in both datasets. From the merged *P* values of the two datasets, we identified the top 12 hotspots in terms of their association strength, and considered them as ‘putative hotspots’. We are interested in the number of hotspots each method recovers from these putative hotspots. The 2008 dataset is of higher quality than the 2005 dataset since it uses a newer version of the array technology. We verified the relative quality of the datasets by comparing the number of *cis*-eQTLs identified in each dataset, which demonstrates that the 2008 dataset is of higher quality. For this reason, we expected that all hotspots originally found in the 2005 dataset that are true effects will be found in the 2008 dataset. Thus hotspots identified in the 2005 dataset but not in the 2008 dataset are likely spurious. 

**Figure 5:**
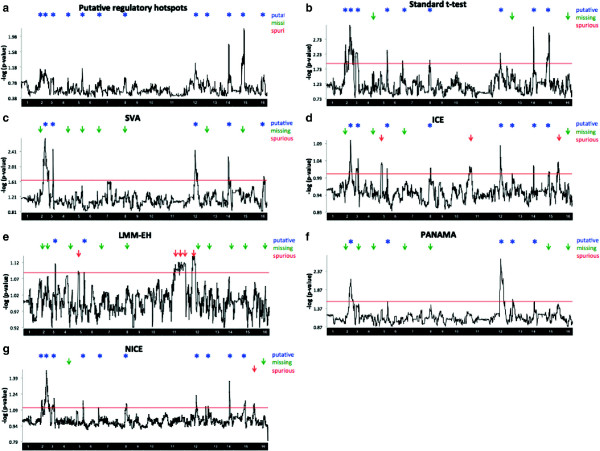
**Putative, missing and spurious hotspots for the methods applied to the yeast dataset generated in 2005 **[2]**. (a)** The average over all genes of the − log of the maximum *P* value of the two yeast datasets for each SNP. **(b)**-**(g)** The average over all genes of the − log *P* value for each SNP for several methods: the standard *t*-test, SVA, ICE, LMM-EH, PANAMA and NICE. Blue asterisks show putative genetic regulatory hotspots predicted from the merged dataset, green arrows show missing hotspots and red arrows show spurious hotspots identified by each method. Red horizontal lines show thresholds used to select significant peaks, which are two standard deviations above the mean. Note that the *t*-test has a distinct advantage in this evaluation because *P* values from the *t*-test were used to determine the putative regulatory hotspots.

We measured the presence of a hotspot by computing the sum of the log *P* values of all of associations of a single marker with the expression level of each gene. This measure, called the hotspot level, identifies hotspots since it captures which SNPs are associated with many gene expression levels. We visualize the hotspot level at the top of our eQTL plots (Figure 4). We used the hotspot level to identify the putative hotspots from the merged *P* values of the two datasets (blue asterisks in Figure 5(a)). 

Our goal in this experiment was to identify the true regulatory hotspots and eliminate the spurious hotspots using the 2005 yeast dataset [2]. We applied each approach to the 2005 data and evaluated the results using knowledge of which hotspots are true hotspots obtained using both the 2005 and the 2008 datasets [2,25]. We computed the hotspot levels for the following methods: the standard *t*-test, SVA, ICE, LMM-EH, PANAMA and NICE (Figure 5). We have annotated the results for each method with blue asterisks, and green and red arrows, which indicate putative genetic regulatory hotspots predicted from the *P* values of merged datasets, missing hotspots and false positive hotspots, respectively. The results show that our method identified all but two of the putative hotspots while only predicting one spurious hotspot (Figure 5(g)). ICE and LMM-EH made several false positive predictions and SVA, LMM-EH and PANAMA missed many hotspots (Figure 5(c) to (f)). We note that the *t*-test has a distinct advantage in this evaluation because *P* values from the *t*-test were used to determine the gold standard (Figure 5(b)) and it is inappropriate to evaluate the *t*-test in terms of sensitivity and FDR estimated from the gold standard. Table 2 summarizes the results. 

**Table 2:** **The number of putative, missing and spurious hotspots for the 2005 yeast data **[2]

**Method**	**Putative hotspots**	**Missing hotspots**	**Spurious hotspots**	**Sensitivity**	**False discovery rate**
*t*-test	9	3	0	0.75	0
SVA	5	7	0	0.42	0
ICE	8	4	3	0.67	0.27
LMM-EH	2	10	5	0.17	0.71
PANAMA	5	7	0	0.42	0
NICE	10	2	1	0.83	0.09

**Figure 6:**
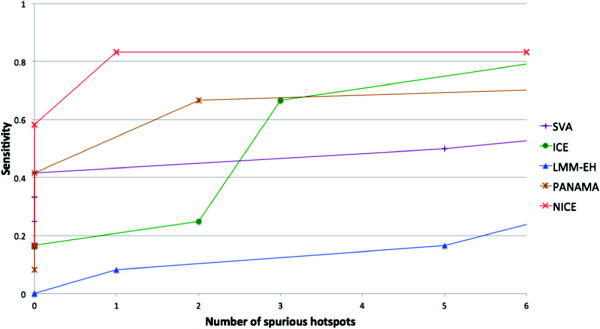
**Sensitivity and the number of spurious hotspots for different thresholds applied to the yeast dataset generated in 2005 **[2]**.** The *x*-axis corresponds to the number of spurious hotspots and the *y*-axis corresponds to the sensitivity. The threshold applied was five standard deviation above the mean (the left and bottom most mark), four standard deviations above the mean, three standard deviations above the mean, etc.

We considered an SNP as a hotspot if its hotspot level was two standard deviations above the mean. We used this criteria because it provides a reasonable threshold to separate hotspots and noisy peaks. Other thresholds were shown to be inappropriate. With lower thresholds, all methods identified not only most of the putative hotspots but also many spurious hotspots. With higher thresholds, all methods missed most of the putative hotspots. Figure 6 is a plot similar to a receiver operating characteristic curve, showing the sensitivity and the number of spurious hotspots for different thresholds. The *x*-axis shows the number of spurious hotspots and the *y*-axis shows the sensitivity. For different thresholds, NICE performed the best. 

**Figure 7:**
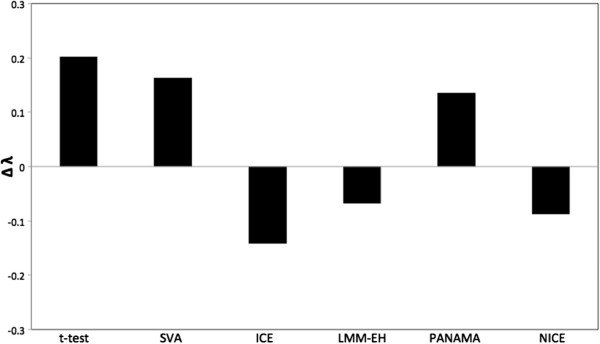
**Inflation factors for different methods for the yeast dataset generated in 2005 **[2]**.** Δλ is defined as 1−λ. FDR, false discovery rate; kb, kilobase.

Figure 7 shows the inflation factors for the methods. The standard *t*-test, SVA and PANAMA show inflation. NICE shows deflation but not as much as ICE and is comparable to LMM-EH. Additional file 1: Figure S1 and Additional file 2: Table S1 show the results of the same analysis from the point of view of analyzing the 2008 data and comparing the hotspots found in the intersection of the 2005 and 2008 datasets. We note that NICE discovered several additional hotspots not identified in the 2005 data, which is expected because the 2008 data is of higher quality in general. Below we show that several of these additional hotspots are likely real genetic effects. 

**Figure 8:**
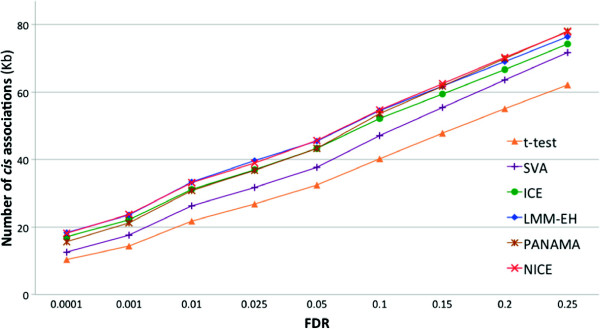
**The number of *cis *associations for the yeast dataset **[2]**.** FDR, false discovery rate; kb, kilobase.

Consistent with previous analyses [18,20,21], to compare the statistical power of the methods, we compared the number of *cis* associations reported by the different methods (Figure 8). NICE was able to identify more *cis* associations than the *t*-test, SVA, ICE or PANAMA, and identified a comparable number of *cis* associations as LMM-EH. This suggest that NICE is not only able to identify true regulatory hotspots but also increases the general sensitivity of the eQTL detection. 

### NICE discovered novel yeast regulatory hotspots

We reanalyzed the 2008 yeast dataset described above using NICE to demonstrate the utility of our approach. The dataset contains expression levels for yeast strains grown in both glucose and ethanol media. In our experiments above, we compared the consistency between the 2005 data and the 2008 data both for yeast grown in glucose. Here we analyzed both conditions in the 2008 data to identify both hotspots in each condition as well as hotspots involved in gene–environment interactions consistent with the previous analyses of this data [25]. To be consistent with the previous analyses, we utilized the method for determining the presence of a hotspot defined in Smith and Kruglyak instead of the metric we used above [25]. We began by dividing the yeast genome into 611 20-kb bins. For each bin, we counted the number of significant *trans* linkages in the bin. Assuming a Poisson process, the number of expected linkages in each bin is the ratio between the number of *trans* linkages and the number of bins. For simplicity, we used the top 3,000 *trans* associations identified by each method yielding λ=4.9. After adjusting for the number of bins using a Bonferroni correction, a bin was considered to have statistically significance (*P*<0.05) if it has >13 linkages. When we identified significant linkages using a *P* value cutoff (*P*<5×10^−5^), we achieved almost the same result.

To compare the regulatory hotspots found by various methods, we first defined 11 putative regulatory hotspots from a collection of independent experiments using the same parental strains grown in glucose [1,27] (Additional file 3: Table S2). Some of these hotspots were expected because of deletions in one of the strains including a hotspot at Chr3:90000 for LEU2 and a hotspot at chr5:110000 for URA3.

We first analyzed the glucose data from Smith and Kruglyak [25]. Table 3 shows the number of known hotspots captured by each method. We see that both the *t*-test and NICE captured nine of the putative regulatory hotspots while ICE captured only eight. Both NICE and ICE captured many more hotspots than the *t*-test and we wanted to know whether these were spurious or real. We analyzed the 2005 dataset using the same definition of regulatory hotspots as in Smith and Kruglyak [25] and found that 2 / 5, 5 / 14 and 8 / 17 of the additional hotspots found by the *t*-test, ICE and NICE, respectively, were replicated. Those additional hotspots that do not overlap with the 2005 dataset could be specific to the 2008 experiment so we further compared them to the ethanol dataset. We found that 3 / 5, 13 / 14 and 15 / 17 of the additional hotspots found by the *t*-test, ICE and NICE, respectively, were replicated in the ethanol experiment. These two results suggest that not only does NICE control for spurious regulatory hotspots, it also discovers more regulatory hotspots that are likely to have a true biological mechanism. 

**Table 3:** **The number of putative, missing and additional hotspots for the 2008 yeast dataset grown in glucose media **[25]

**Method**	**Putative**	**Missing**	**Additional**	**Glucose shared**	**Ethanol shared**
*t*-test	9	2	5	2	3
ICE	8	3	14	5	13
NICE	9	2	17	8	15

The last two columns show the number of glucose-shared and ethanol-shared hotspots for the additional hotspots compared to the glucose dataset generated in 2005 [2] and the ethanol dataset generated in 2008 [25].

One of the additional hotspots NICE found to be shared between ethanol and glucose is at Chr7:380000. However, from the *t*-test results, this hotspot appears to be ethanol specific. We further confirmed that this hotspot was also found by NICE for the 2005 data, suggesting that it is likely to be a real hotspot that is not condition specific. The two possible candidate genes are RPB9 and MNP1 since the regulatory hotspot is linked in *cis* to the expression of both of these genes at *P* values of 6.8×10^−6^ and 2.2×10^−4^, respectively. RPB9 is a RNA polymerase II subunit that is crucial for transcription fidelity while MNP1 is a putative mitochondrial ribosomal protein that is required for respiratory growth. NICE also found an additional hotspot at Chr14:1360000 for the glucose data from both 2005 and 2008, but which is absent from the ethanol data suggesting that it is a glucose-specific hotspot. The *t*-test did not find this hotspot for either glucose dataset. The closest gene is APT2, which is an apparent pseudogene that is not expressed in normal conditions. Interestingly, with our data, we found a strong association between Chr14:1360000 and APT2 in *cis* at *P*=2.3×10^−17^, suggesting that this gene might be functional in a glucose-dependent way.

## Discussion

In this paper, we present a novel approach, NICE, for identifying true genetic regulatory hotspots while eliminating spurious hotspots caused by confounding factors. We leveraged the insight that confounding factors are likely to affect the majority of genes, while genetic effects are likely to affect only a smaller subset. This insight allowed our approach to distinguish between true and spurious regulatory hotspots. Our approach is related to previous methods that correct for confounding factors, such as ICE or SVA, which model the global correlation structure and use this structure to correct the association statistics to eliminate the effect of any confounding factors affecting the association statistic. NICE uses only a subset of genes predicted not to be part of the true genetic hotspot to model the global correlation structure between expression levels to correct for confounding factors, which eliminates the confounding factors but preserves true hotspots. We compared several previous approaches [18-21] with both simulated and real datasets, and demonstrated that our method achieved higher or comparable statistical power when identifying associations while correcting for confounding factors.

While our approach, NICE, extends the mixed model approach that ICE [18] used, in principle, the basic idea behind our approach can be applied to other approaches for correcting for expression heterogeneity such as the SVA approach based on singular value decomposition [19]. In this method, for each SNP, a separate singular value decomposition would be computed only taking into account genes that are predicted not to be part of a genetic hotspot. Similarly, the techniques used in LMM-EH [20] can also be adapted in this framework to incorporate multiple variance components to correct for population structure and also correct for bias in estimating the global correlation structure in the presence of population structure.

Our method is based on the assumption that confounding factors are likely to affect the majority of genes, while genetic effects are likely to affect only a subset of the genes. While our approach is an improvement over current methods, in some cases this assumption may be violated, for example, slightly different growth temperatures between batches may result in a specific subset of genes being differentially expressed (e.g. heat shock, cell cycle regulators, etc.). In these cases, our approach would be unable to distinguish those confounding effects from real genetic effects. An additional challenge in eQTL studies is correcting for multiple testing. Possible approaches for multiple testing correction are applying permutation tests or FDRs. Unfortunately, when confounding is present, the confounding causes a violation of the basic assumption necessary for these approaches, which is that the individuals in the sample are independent and identically distributed. Shared confounding factors induces complex dependencies among the gene expression patterns of individuals and complicates multiple testing. How to correct for multiple testing in the presence of confounding is a fundamental problem and a promising avenue of future work, which is beyond the scope of this paper.

## Conclusions

In this paper, we introduce a novel statistical method that effectively eliminates spurious regulatory hotspots resulting from various confounding factors while retaining genuine hotspots resulting from true genetic effects. In simulations, our method perfectly segregates genuine and spurious hotspots. We validate our method on yeast data where the locations of true genetic hotspots are known through concordance between replicated datasets. Our method achieves greater sensitivity (83%) in detecting concordant hotspots compared to previous methods while retaining a low false discovery rate. In addition to detecting regulatory hotspots, our method identifies more or a comparable number of cis-eQTLs than other methods. We further apply our method to yeast data grown in different conditions to identify gene-by-environment interactions and show that our method discovers novel yeast regulatory hotspots that are likely to have a true biological mechanism.

## Materials and methods

### Generative model

We assume the following linear mixed model as the generative model of the expression levels,

(1) Y=μ+Xβ+u+e

Let *n* be the number of individuals, *m* the number of genes and *l* the number of SNPs. *Y* is an *n*×*m* matrix of the gene expression values, *μ* is an *n*×*l* matrix of the means of expression levels of individuals, *X* is an *n*×*l* matrix with SNPs encoded by 0 and 1 for haploid and 0, 1 and 2 for diploid, *β* is an *l*×*m* matrix for their coefficients, and *u* and *e* are *n*×*m* matrices with multivariate normal random variables sampled from $N(0,\sigma_{g}^{2}H)$ and $N(0,\sigma_{e}^{2}I)$ accounting for the confounding effects and random errors. Here, *H* is an *n*×*n* covariance matrix that explains the intersample correlation structure induced by confounders and *I* is an *n*×*n* identity matrix. $\sigma_{g}^{2}$ and $\sigma_{e}^{2}$ are coefficients of the two variance components.

### eQTL mapping

Based on our generative model, equation (1), we map eQTL as follows. To test the effect of SNP *j* on the expression level of gene *i*, we assume the model

(2) $$y_{i}=\mu_{i}+x_{j}\beta_{\text{ij}}+u_{i}+\varepsilon_{\text{ij}}$$

where *y*_*i*_ is a size *n* vector denoting gene expression levels of individuals, *μ*_*i*_ is a size *n* vector denoting the mean of expression levels of individuals, *x*_*j*_ is a size *n* binary vector denoting SNPs of individuals, $u_{i}∼N(0,\sigma_{g}^{2}H)$ are confounding effects, and $\varepsilon_{\text{ij}}∼N(0,\sigma_{e}^{2}I)$ are residual errors. The null hypothesis that we want to test is *β*_*ij*_=0. Typically, *H* is defined or estimated before the eQTL mapping. Given an estimated $\hat{H}$, we use the efficient mixed-model association (EMMA) C package [28] to estimate efficiently the variance components ($\sigma_{g}^{2}$ and $\sigma_{e}^{2}$). We use the F test as previously suggested on the basis of REML (Restricted Maximum Likelihood) estimates of variance components [28-30]. The challenge in this model is how to estimate $\hat{H}$ that is close to the true covariance structure of confounding, *H*.

### ICE eQTL mapping

The ICE eQTL mapping approach [18] utilizes a global intersample correlation generated from all genes to estimate *H*. The global intersample correlation matrix for an expression dataset is generated as follows. Let *Y* be an *m*×*n* expression matrix with *n* individuals for *m* genes. Let *μ*_*i*_,*σ*_*i*_ be the mean and standard deviation of expression values of the *i*th genes (*Y*_*i*1_,*Y*_*i*2_,...,*Y*_*in*_). Let *Z* be an *m*×*n* matrix with each element *Z*_*ij*_=(*Y*_*ij*_−*μ*_*i*_)/*σ*_*i*_. The intersample correlation matrix is defined as the covariance matrix of *Z*, $\hat{H}=\mathrm{Cov}(Z)$. The estimated intersample correlation matrix $\hat{H}$ is then used in the linear mixed model in equation (2) to correct for the confounding effects.

### NICE eQTL mapping

We propose a new eQTL mapping approach called NICE eQTL mapping. NICE builds upon the framework of ICE eQTL mapping but uses a more refined strategy to estimate *H*, the covariance matrix of confounding effects. The primary limitation of ICE is that it uses the global intersample correlation generated from all genes. If there exists a regulatory hotspot that affects many genes, ICE will over-correct for the confounding and remove the associations to the regulatory hotspot. To overcome this challenge, we must use the genes that are only affected by the confounding but not by the regulatory hotspots to estimate *H*. It turns out that segregating these two groups of genes is a highly challenging computational problem.

#### Assumptions

We assume that confounding affects the global correlation structure of the gene expressions thereby affecting most of the genes. This is a standard assumption consistent with previous approaches. We then assume that true regulatory hotspots affect only a subset of the genes. This assumption will be invalid only if a hotspot affects most of the genes, which will be unlikely in practice. Our goal is to separate the genes affected by the true genetic effects from the genes affected only by the confounding. If we can successfully separate them, we will be able to estimate *H* more accurately using the genes affected only by the confounding.

To this end, we make an assumption that the effect size of genetic effects is greater than the magnitude by which the confounding affects the expression levels. That is, we assume that the genes with true genetic effects tend to have more significant results than the other genes affected only by the confounding. This assumption may not be true if the genetic effects are small and the confounding is severe, but in such cases, the noisy data will be highly challenging and in this paper we will ignore such cases. Additionally, we assume that the true genetic effects of regulatory hotspot may have a structure. For example, the hotspot may be related to an enhancer element upregulating many genes, in which case the mean of the effect will be nonzero. Ideally, we would want to use such structures to discriminate the true genetic effects from confounding.

#### Bayesian framework

A Bayesian framework fits well with our purpose of separating the genes with true genetic effects from the genes with only confounding effects, because it gives each gene a posterior probability that the genetic effect will exist or not. Given an SNP that we want to test, we first apply the standard *t*-test between the SNP and all genes to obtain the effect sizes and standard errors of the SNP effect with respect to all genes. Let *β*_*i*_ be the estimated effect size of the SNP to gene *i* and let *V*_*i*_ be the variance of it. We assume a model that

$$P(\beta_{i}|\text{no genetic effect})=N(\beta_{i};0,V_{i})$$

and

$$P(\beta_{i}|\text{genetic effect})=N(\beta_{i};\mu ,V_{i})$$

Note that a few simplifications are employed in this model. First, based on our assumption that the confounding effects are sufficiently smaller than the genetic effects, we approximated the confounding effects as zero. Second, to capture the possible structure within genetic effects, we employed the mean term *μ*. Although this is a simplified model, we found that this approach can capture the majority of the genes affected by the genetic effects, which turns out to be sufficient for our purpose of finding an accurate *H*.

We assume a prior for the effect size

$$\mu ∼N(0,\sigma^{2})$$

Let *T*_*i*_ be a random variable, which is 1 if gene *i* is affected by the genetic effect of the SNP of interest and 0 otherwise. Let *π* be the prior probability that each gene is affected by the genetic effect such that

$$P(T_{i}=1)=\pi ,\;i=1,\mathrm{...},m$$

Then we assume a beta prior on *π*

$$\pi ∼\mathrm{Beta}(\alpha_{1},\alpha_{2})$$

Let *T*=(*T*_1_,...,*T*_*m*_) be the vector indicating the existence of a genetic effect in all genes. Let $\vec{\beta }=(\beta_{1},\ldots ,\beta_{m})$. Our goal is to estimate the posterior probability that the genetic effect exists for each gene *i*, namely

$$P(T_{i}=1|\vec{\beta })$$

Notice that *T* can have 2^*m*^ different values. Let $U=\{t_{1},\ldots ,t_{2^{m}}\}$ be the set of those values. By Bayes’ theorem,

(3) $$\;\begin{array}{ll} P(T_{i}=1|\vec{\beta }) & =\frac{P(\vec{\beta }|T_{i}=1)P(T_{i}=1)}{P(\vec{\beta }|T_{i}=0)P(T_{i}=0)+P(\vec{\beta }|T_{i}=1)P(T_{i}=1)}\\ & =\frac{\underset{t\in U_{i}}{\sum }P(\vec{\beta }|T=t)P(T=t)}{\underset{t\in U}{\sum }P(\vec{\beta }|T=t)P(T=t)} \end{array}$$

where *U*_*i*_ is a subset of *U* whose elements’ *i*th value is 1. Thus, we should calculate for each *t* the posterior probability of *T*,

$$g(t)=P(\vec{\beta }|T=t)P(T=t)\propto P(T=t|\vec{\beta })$$

consisting of the probability of $\vec{\beta }$ given *T* and the prior probability of *T*.

#### Connection to meta-analytic approach

It turns out that our Bayesian model for eQTL mapping is equivalent to a meta-analysis model although their contexts are different. In a meta-analysis of genetic association studies that combines multiple independent studies, if there exists heterogeneity, which refers to the differences in effect sizes of studies [31], it is challenging to predict which study has an effect and which study does not. Thus, the problem of finding studies with an effect is essentially equivalent to the problem of finding genes having genetic effects in our context. Recently, we have developed an efficient method to solve this problem in the context of meta-analysis [24]. Here we adapt this approach to calculate the posterior probability of *T*. We briefly describe below how we can calculate *g*(*t*).

*g*(*t*) consists of the prior probability of *T* and the probability of $\vec{\beta }$ given *T*. The prior probability of *T* is

$$\begin{array}{ll} P(T=t) & =\int_{-\infty }^{\infty }P(T=t|\pi )p(\pi )\mathrm{d\pi}\\ & =\int_{-\infty }^{\infty }\pi^{\left|t\right|}{\left(1-\pi \right)}^{m-|t|}p(\pi )\mathrm{d\pi}\\ & =\int_{-\infty }^{\infty }\;\pi^{\left|t\right|}{\left(\;1-\pi \;\right)}^{m-|t|}\frac{1}{B(\alpha_{1},\alpha_{2})}\pi^{\alpha_{1}-1}{\left(\;1-\pi \;\right)}^{\alpha_{2}-1}\mathrm{d\pi}\\ & =\frac{B(|t|+\alpha_{1},m-|t|+\alpha_{2})}{B(\alpha_{1},\alpha_{2})} \end{array}$$

where |*t*| is the number of 1’s in *t* and *B* is the beta function.

The probability of $\vec{\beta }$ given *T* is

(4) $$\begin{array}{ll} P(\vec{\beta }|T=t) & =\int_{-\infty }^{\infty }\underset{i\in t_{0}}{\prod }N(\beta_{i};0,V_{i})\underset{i\in t_{1}}{\prod }N(\beta_{i};\mu ,V_{i})p(\mu )\mathrm{d\mu}\\ & =\underset{i\in t_{0}}{\prod }N(\beta_{i};0,V_{i})\int_{-\infty }^{\infty }\underset{i\in t_{1}}{\prod }N(\beta_{i};\mu ,V_{i})p(\mu )\mathrm{d\mu} \end{array}$$

where *t*_0_ are the indices of 0 in *t* and *t*_1_ are the indices of 1 in *t*. We can analytically work out the integration to obtain

$$\begin{array}{lll} \int_{-\infty }^{\infty }\underset{i\in t_{1}}{\prod }N(\beta_{i};\mu ,V_{i})p(\mu )\mathrm{d\mu} & =\bar{C}\cdot N(\bar{\beta };0,\bar{V}+\sigma^{2})\; & \; \end{array}$$

where

$$\bar{\beta }=\frac{\sum_{i}W_{i}\beta_{i}}{\sum_{i}W_{i}}\;\text{and}\;\bar{V}=\frac{1}{\sum_{i}W_{i}}$$

where $W_{i}=V_{i}^{-1}$ is the inverse variance or precision. The summations are all with respect to *i*∈*t*_1_. $\bar{C}$ is a scaling factor such that

$$\begin{array}{c} \bar{C}\;=\;\frac{1}{{\left(\sqrt{2\pi }\right)}^{m-1}}\sqrt{\frac{\prod_{i}W_{i}}{\sum_{i}W_{i}}}\exp\;\left\{\;-\frac{1}{2}\left(\;\sum_{i}W_{i}\beta_{i}^{2}-\frac{{\left(\sum_{i}W_{i}\beta_{i}\right)}^{2}}{\sum_{i}W_{i}}\;\right)\;\right\} \end{array}$$

See Han and Eskin [24] for the details of the derivation. As a result, we can calculate *g*(*t*) for every *t*.

#### Markov chain Monte Carlo

Although we calculate *g*(*t*) for each *t*, it is impractical to perform an exact calculation of $P(T_{i}=1|\vec{\beta })$ in equation (3) since we have a large number of genes. Thus, we use the following Markov chain Monte Carlo (MCMC) method [24]: 

1. Start from a random *t*.

2. Choose a next *t*, *t*^′^, based on the moves defined below.

3. If *g*(*t*)<*g*(*t*^′^), move to *t*^′^. Otherwise, move to *t*^′^ with probability *g*(*t*^′^)/*g*(*t*).

4. Repeat from step 2.

The set of moves we use for choosing *t*^′^ is {*M*_1_,*M*_2_,…,*M*_*m*_}∪{*M*_shuffle_}. *M*_*i*_ is a simple flipping move of *T*_*i*_ between 0 and 1. *M*_shuffle_ is a move that shuffles the values of *T*. At each step, we randomly choose a move from this set assuming a uniform distribution. Other moves can also be used, such as moves based on Bayes’ factors. We allow *n*_*B*_ burn-in and sample *n*_*S*_ times. After sampling, *n*_*S*_ samples gives us an approximation of the distribution over *g*(*t*), which subsequently gives the approximations of equation (3). Calculating the posterior probability is the most computationally intensive part of NICE relative to ICE [18] with respect to the running time. By using MCMC, we make dramatic reductions in computational cost, which allows NICE to scale to large datasets.

#### NICE intersample correlation matrix

After we calculate the posterior probability that a gene is affected by the true genetic effect of the SNP, we select genes with a probability less than a threshold *η*=0.5. This set of genes represents the genes that are putatively affected only by the confounding. Thus, we use this set of genes to build the intersample correlation matrix ${\hat{H}}_{\text{NICE}}$. Then we apply ${\hat{H}}_{\text{NICE}}$ to the linear mixed model (2) to correct for the confounding in our eQTL mapping. The reason why we choose *η*=0.5 as a threshold is because we want to find a subset of genes approximately without genetic effects. Although it is ideal to select all the genes without genetic effects, any subset of those genes is likely to capture the global correlation structure as shown in Figure 1(c), and is enough to correct for confounding effects. We choose genes that have an effect with less than 50% chance to select genes that are putatively affected only by the confounding effect. However, we find that unless the threshold is extreme (e.g. *η*≤0.1 or *η*≥0.9), all thresholds yield similar results and the result is robust to the parameter (Additional file 4: Figure S2). We count the number of genes selected by NICE using the posterior probability with threshold *η*=0.5 applied to the yeast data generated in 2005 [2] (blue dots in Additional file 5: Figure S3). Except for the putative hotspots, mostly, NICE uses the majority of the genes to build the intersample correlation matrix ${\hat{H}}_{\text{NICE}}$ similar to ICE [18].

#### Implementation

To calculate the posterior probability in equation (3), we used METASOFT [31] with prior parameters, *σ*=0.05, *α*_1_=1 and *α*_2_=5. We used *σ*=0.05 by assuming a small effect size. However, an effect size of up to 0.4, which is a possible choice as a large effect size in a genome-wide association study [32,33], did not affect the results significantly (Additional file 6: Figure S4). We assume that confounding affects most of the genes while true regulatory hotspots affect only a subset of the genes. Based on the assumption, we assume that 20% of the genes have *trans* effects for our prior (*α*_1_/*α*_2_=0.2). We used *α*_1_=1 and *α*_2_=5 to give a diffuse distribution. In practice, changing the *α*_1_ and *α*_2_ priors gives similar results as changing the threshold *η* (data not shown). As shown in Additional file 4: Figure S2, the results are robust unless the threshold/priors are too extreme. If one does not have prior information about a dataset, we suggest using these default priors as they are based on our model assumption. We used *n*_*B*_=1,000 burn-in and *n*_*S*_=1,000,000 sampling in MCMC. We selected genes with posterior probability less than *η*=0.5. If less than 1% of the genes were selected to calculate the covariance matrix, we used the standard *t*-test instead of NICE.

### *P* value based approach

Instead of using the posterior probability described in the previous sections, here we show whether a more standard test statistic, such as the *P* value from the standard *t*-test, could be used for selecting genes without genetic effects to estimate the intersample correlation matrix *H*. We use the *P* value for selecting genes without genetic effects in the following approach. For each SNP, we first order the genes based on the *P* value obtained using the standard *t*-test, { *g*_1_,*g*_2_,…,*g*_*m*_}, where *g*_1_ is the gene with the largest *P* value and *g*_*m*_ is the gene with the smallest *P* value when there are *m* genes. Then we select the first *x*% of the ordered genes { *g*_1_,*g*_2_,…,*g*_*xm*/100_} as the genes without genetic effects and use the expression levels of those genes to estimate *H*. The following processes are the same as those used for NICE. Let us say *α* is the percentage of genes that have *trans* effects on a *trans*-regulatory hotspot. When we apply this approach for various simulated datasets with different *α*, *x*=100−*α* gives the best estimation of *H* for correcting for the confounding effects but retaining the true genetic effects (here we show only the case when *α*=20). However, when we use fewer (*x*<100−*α*) or more (*x*>100−*α*) genes, we fail to remove confounding effects or fail to retain the true genetic effects, respectively. Additional file 7: Figure S5 shows eQTL maps for when this approach was applied to our simulated data. Our simulated data has *trans* effects on 20% of the genes (*α*=20), in other words, 80% of genes do not have *trans* effects. Therefore, when we use 80% (*x*=80) of the genes to build the intersample correlation matrix *H*, we are able to correct for the confounding effects but retain the genetic effects. However, when we use fewer of the genes, e.g. 60% (*x*=60), or more of them, e.g. 99% (*x*=99), we either fail to remove confounding effects or fail to retain the true genetic effects, respectively. Unfortunately, we do not know how many genes have *trans* effects for each marker in advance. Moreover, we note that this approach creates many spurious associations other than the ones induced by confounding effects. For example, many horizontal lines appear in the eQTL map (Additional file 7: Figure S5(a)). This is because when we select *x*% of genes with the largest *P* values, some of the selected genes are shared between many SNPs and this creates spurious associations between the shared genes and the SNPs.

We also applied this approach to the yeast dataset generated in 2005 [2] using 10%, 30%, 50%, 70% and 90% of the genes. As a result, it missed many putative hotspots and made many false positive predictions (Additional file 8: Figure S6). Thus, we conclude that the *P* value is ineffective for selecting genes without genetic effects. On the other hand, the posterior probability that we use for NICE is robust as the value of *η* neither has a significant influence on the results nor is specific to the datasets.

### Simulated dataset

We generated a simulated dataset for 1,000 genes, 1,000 SNPs, and 100 samples based on our generative model, equation (1), with *σ*_*g*_=0.9 and *σ*_*e*_=0.1. Assuming haploidy, the SNPs were encoded by 0 and 1 and randomly generated with a minor allele frequency of 30%. A batch effect was simulated as a confounding effect where expression levels in the first half of the samples were correlated with each other, but not correlated with the second half of the samples, and vice versa. Five randomly selected *trans*-regulatory hotspots were simulated and for each of them, 20% of the genes had *trans* effects of size 0.4 where half had positive effects and the other half had negative effects. The *cis* effect was simulated with a size of 0.5.

### Yeast datasets

We evaluated our method using replicate gene expression datasets. We used two versions of a yeast dataset produced 3 years apart at different locations using different microarray platforms. The first dataset [2] was generated in 2005, of which 6,138 probes and 2,956 genotyped loci in 112 segregants were used. The second dataset [25] was generated in 2008, of which 6,138 probes and 2,956 genotyped loci in 109 segregants were used. We classified the eQTL as *cis*-acting when the location of an SNP and the location of a probe were within 50 kb. We calculated the number of *cis*-eQTLs for different FDRs where the FDRs were calculated using the *q* value function of R.

### Running previous methods

For running previous methods, SVA [19], ICE [18], LMM-EH [20] and PANAMA [21], we downloaded the program available from the authors and ran it using default options. For running SVA, the ‘two-step’ method was used. For running LMM-EH, eLMM v1.2 was used for generating the covariance matrix *K*_*EH*_ and FaST-LMM v2.05 [34] was used for calculating the associations. For running eLMM, the ICE covariance matrix was used for the initial *K*_*EH*_. The EM (Expectation Maximization) steps for each full iteration was set to 3 and 10 ∼ 20 number of total iterations was applied. eLMM includes LMM-EH-PS, which corrects for confounding factors as well as population structure. We used LMM-EH instead of LMM-EH-PS because neither our simulated nor yeast dataset contained a population structure. In addition, LMM-EH-PS failed to run on our Windows machine with 1.73 GHz Intel Core i7 CPU and 4 GB RAM. Nicolo Fusi, the author of PANAMA, helped with running this program.

## Abbreviations

eQTL: expression quantitative trait loci; FDR: false discovery rate; ICE: Intersample Correlation Emended; kb: kilobase; MCMC: Markov chain Monte Carlo; NICE: Next-generation Intersample Correlation Emended; SNP: single nucleotide polymorphism.

## Competing interests

The authors declare that they have no competing interests.

## Authors’ contributions

All authors designed the methods and experiments, and jointly performed the analysis. JWJJ implemented the methods. All authors discussed the results and contributed to the writing of the manuscript. All authors read and approved the final manuscript.

## Supplementary Material

Additional file 1 **Figure S1.** Putative, missing and additional hotspots for the standard *t*-test, SVA, ICE, LMM-EH, PANAMA and NICE applied to the yeast dataset generated in 2008 [25]. **(a)** The average over all genes of the −log of the maximum *P* value of the two yeast datasets for each SNP. **(b)-(g)** The average over all genes of the −log*P* value for each SNP for the standard *t*-test, SVA, ICE, LMM-EH, PANAMA and NICE. Blue asterisks show putative genetic regulatory hotspots predicted from merged dataset, green arrows show missing hotspots and red arrows show additional hotspots. Red horizontal lines show the thresholds used to select significant peaks, which are two standard deviations above the mean. Note that the *t*-test has a distinct advantage in this evaluation because *P* values from the *t*-test were used to determine the putative regulatory hotspots. Click here for file

Additional file 2 **Table S1.** The number of putative, missing and additional hotspots identified by the different methods applied to the yeast data generated in 2008 [25]. Click here for file

Additional file 3 **Table S2.** List of putative hotspots. We defined 11 putative regulatory hotspots from a collection of independent experiments using the same parental strains grown in glucose [1,27]. Click here for file

Additional file 4 **Figure S2.** eQTL maps for NICE using different thresholds for simulated data. **(a)-(c)** Thresholds of *η*=0.3, *η*=0.5 and *η*=0.7, respectively. Blue arrows show the locations of real genetic regulatory hotspots. Click here for file

Additional file 5 **Figure S3.** Number of genes used to build ${\hat{H}}_{\text{NICE}}$ for the yeast dataset generated in 2005 [2]. The bottom plot shows hotspot levels for NICE as in Figure 5(g). The blue dots above the hotspot levels show the number of genes selected by NICE using a posterior probability less than a threshold *η*=0.5. Click here for file

Additional file 6 **Figure S4.** eQTL maps of NICE using different *σ* values applied to simulated data. **(a)-(c)***σ*=0.05, *σ*=0.2 and *σ*=0.4, respectively. Blue arrows show the locations of real genetic regulatory hotspots. The results from NICE are robust to the prior *σ*. Click here for file

Additional file 7 **Figure S5.** eQTL maps when *P* values are used for selecting genes without genetic effects to build *H* for simulated data. **(a),(b),(c)** eQTL maps when 60% (*x*=60), 80% (*x*=80) and 99% (*x*=99) of the genes with the largest *P* values were selected, respectively. The simulated data has *trans* effects for 20% of the genes for each *trans*-regulatory hotspot. Blue arrows show the locations of real genetic regulatory hotspots. Click here for file

Additional file 8 **Figure S6.** Putative, missing and spurious hotspots when *P* values are used to build *H* for the yeast dataset from 2005 [2]. **(a)** Putative hotspots as in Figure 5(a). **(b) to (f)** eQTL maps when 10% (*x*=10), 30% (*x*=30), 50% (*x*=50), 70% (*x*=70) and 90% (*x*=90) of the genes with the largest *P* values are selected, respectively. Click here for file
