# Supplementary figures and images for: Effectively identifying regulatory hotspots while capturing expression heterogeneity in gene expression studies
Source: Genome Biol. 2014 Apr 7;15(4):r61. doi: 10.1186/gb-2014-15-4-r61 (PMC4053820; doi:10.1186/gb-2014-15-4-r61)

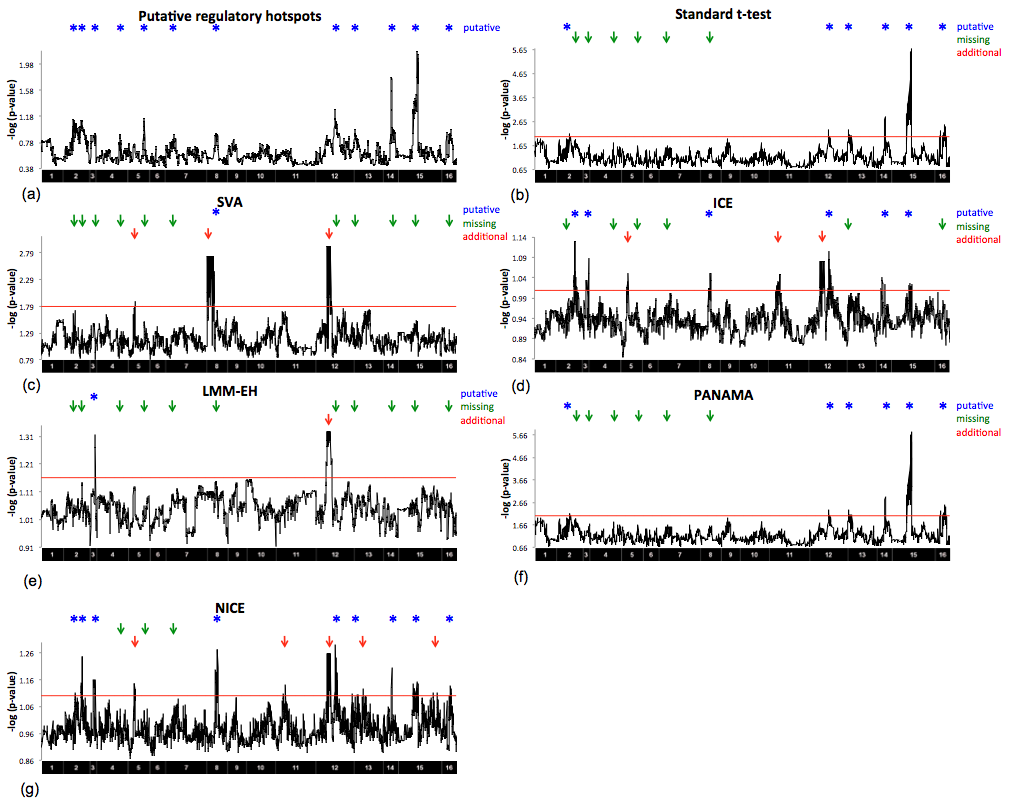

Supplement: Additional file 1 — Figure S1. Putative, missing and additional hotspots for the standard t-test, SVA, ICE, LMM-EH, PANAMA and NICE applied to the yeast dataset generated in 2008 [25]. (a) The average over all genes of the −log of the maximum P value of the two yeast datasets for each SNP. (b)-(g) The average over all genes of the −logP value for each SNP for the standard t-test, SVA, ICE, LMM-EH, PANAMA and NICE. Blue asterisks show putative genetic regulatory hotspots predicted from merged dataset, green arrows show missing hotspots and red arrows show additional hotspots. Red horizontal lines show the thresholds used to select significant peaks, which are two standard deviations above the mean. Note that the t-test has a distinct advantage in this evaluation because P values from the t-test were used to determine the putative regulatory hotspots. [file gb-2014-15-4-r61-S1.png]

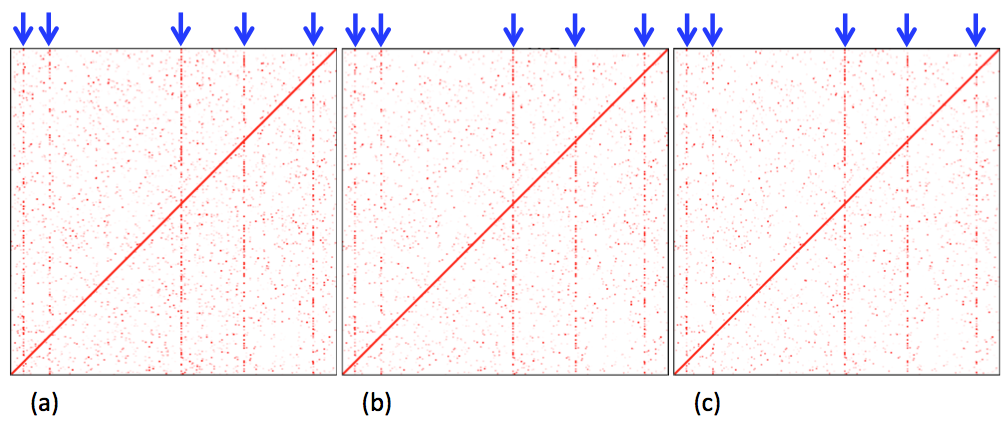

Supplement: Additional file 4 — Figure S2. eQTL maps for NICE using different thresholds for simulated data. (a)-(c) Thresholds of η=0.3, η=0.5 and η=0.7, respectively. Blue arrows show the locations of real genetic regulatory hotspots. [file gb-2014-15-4-r61-S4.png]

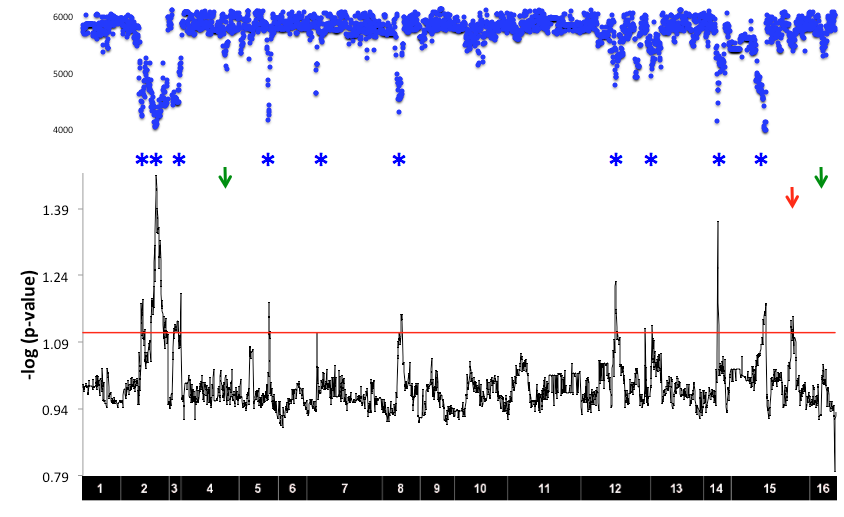

Supplement: Additional file 5 — Figure S3. Number of genes used to build H^NICE for the yeast dataset generated in 2005 [2]. The bottom plot shows hotspot levels for NICE as in Figure 5(g). The blue dots above the hotspot levels show the number of genes selected by NICE using a posterior probability less than a threshold η=0.5. [file gb-2014-15-4-r61-S5.png]

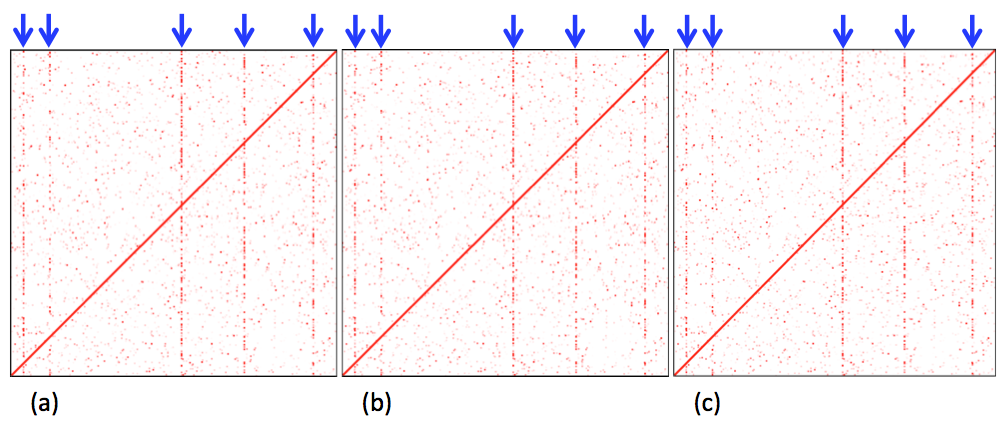

Supplement: Additional file 6 — Figure S4. eQTL maps of NICE using different σ values applied to simulated data. (a)-(c)σ=0.05, σ=0.2 and σ=0.4, respectively. Blue arrows show the locations of real genetic regulatory hotspots. The results from NICE are robust to the prior σ. [file gb-2014-15-4-r61-S6.png]

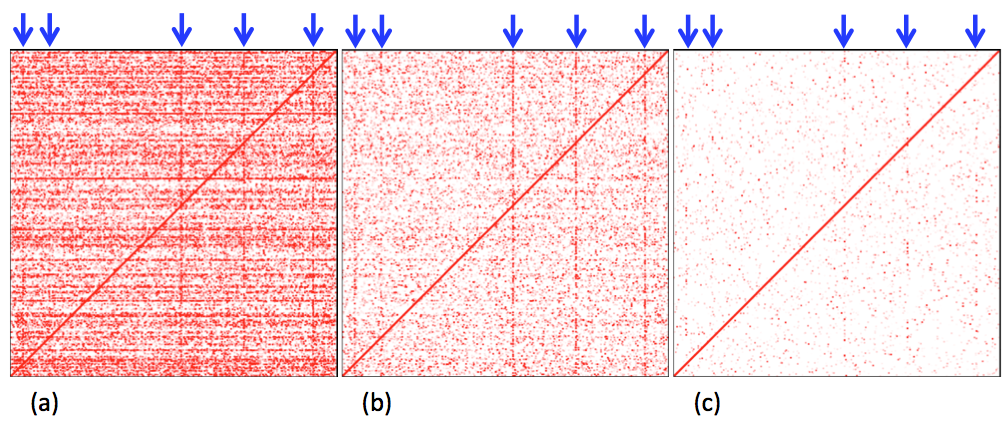

Supplement: Additional file 7 — Figure S5. eQTL maps when P values are used for selecting genes without genetic effects to build H for simulated data. (a),(b),(c) eQTL maps when 60% (x=60), 80% (x=80) and 99% (x=99) of the genes with the largest P values were selected, respectively. The simulated data has trans effects for 20% of the genes for each trans-regulatory hotspot. Blue arrows show the locations of real genetic regulatory hotspots. [file gb-2014-15-4-r61-S7.png]

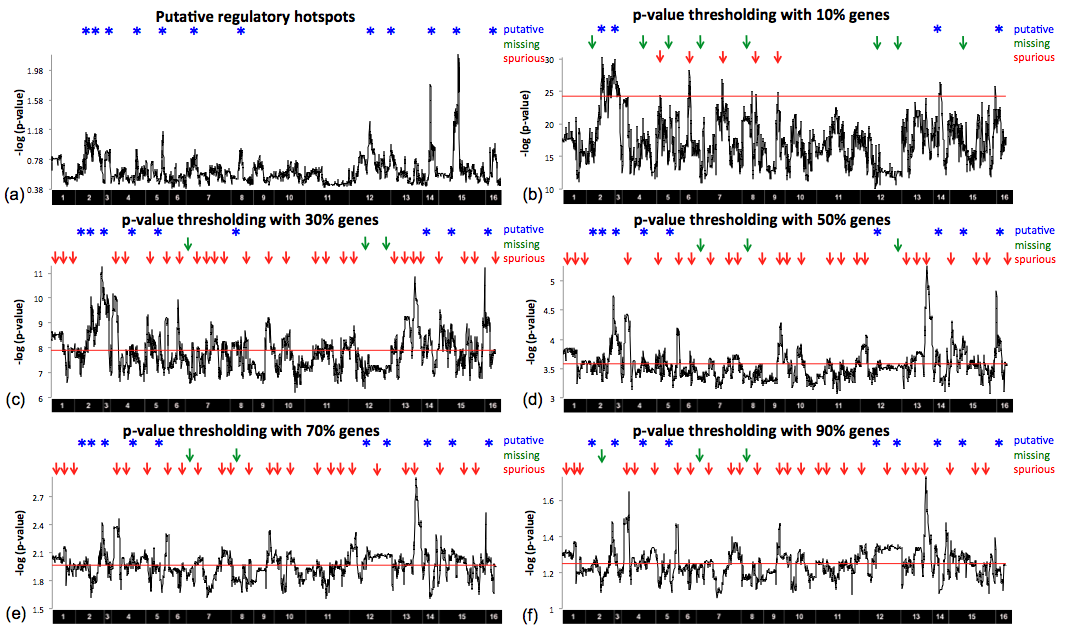

Supplement: Additional file 8 — Figure S6. Putative, missing and spurious hotspots when P values are used to build H for the yeast dataset from 2005 [2]. (a) Putative hotspots as in Figure 5(a). (b) to (f) eQTL maps when 10% (x=10), 30% (x=30), 50% (x=50), 70% (x=70) and 90% (x=90) of the genes with the largest P values are selected, respectively. [file gb-2014-15-4-r61-S8.png]
