# Supplementary material for: Effectively identifying regulatory hotspots while capturing expression heterogeneity in gene expression studies
Source: Genome Biol. 2014 Apr 7;15(4):r61. doi: 10.1186/gb-2014-15-4-r61 (PMC4053820; doi:10.1186/gb-2014-15-4-r61)
Supplement: Additional file 2 — Table S1. The number of putative, missing and additional hotspots identified by the different methods applied to the yeast data generated in 2008 [25]. [file gb-2014-15-4-r61-S2.pdf]

Method	Putative hotspots	Missing hotspots	Additional hotspots
t-test	6	6	0
SVA	1	11	3
ICE	6	6	3
LMM-EH	1	11	1
PANAMA	6	6	0
NICE	9	3	5
