# Supplementary material for: Effectively identifying regulatory hotspots while capturing expression heterogeneity in gene expression studies
Source: Genome Biol. 2014 Apr 7;15(4):r61. doi: 10.1186/gb-2014-15-4-r61 (PMC4053820; doi:10.1186/gb-2014-15-4-r61)
Supplement: Additional file 3 — Table S2. List of putative hotspots. We defined 11 putative regulatory hotspots from a collection of independent experiments using the same parental strains grown in glucose [1,27]. [file gb-2014-15-4-r61-S3.pdf]

Chromosome	Position	Gene
2	380000	unknown
2	550000	AMN1
3	90000	LEU2
3	200000	MAT
5	110000	URA3
8	90000	GPA1
12	670000	HAP1
12	1000000	SIR3
14	480000	unknown
15	180000	unknown
15	580000	CAT5
